# *Six2creFrs2α* knockout mice are a novel model of renal cystogenesis

**DOI:** 10.1038/srep36736

**Published:** 2016-11-17

**Authors:** Pawan Puri, Daniel Bushnell, Caitlin M. Schaefer, Carlton M. Bates

**Affiliations:** 1 Division of Nephrology, Department of Pediatrics, University of Pittsburgh School of Medicine, Pittsburgh, PA, USA; 2 Children’s Hospital of Pittsburgh of UPMC, Pittsburgh, PA, USA

## Abstract

*Six2cre*-mediated deletion of Frs2α (*Six2creFrs2αKO*), a major fibroblast growth factor receptor (Fgfr) docking protein in mouse nephron progenitors results in perinatal renal hypoplasia; however, postnatal *Six2creFrs2αKO* kidneys develop cysts. We sought to determine the pathogenesis of *Six2creFrs2αKO* cyst formation. We performed histological assays, Western blots, and quantitative PCR (qPCR). While embryonic day (E) 18.5 *Six2Frs2αKO* kidneys were hypoplastic and not cystic, postnatal day (P) 7 mutants had proximal tubular-derived cysts that nearly replaced the renal parenchyma by P21. Mutants had high proximal tubular proliferation rates and interstitial fibrosis, similar to known polycystic kidney disease (PKD) models. *Six2creFrs2αKO* kidneys also had upregulation of Wnt/βcatenin signaling, macrophage infiltration and chemokine production (e.g. ectopic Ccl2 in non-dilated proximal tubules), and augmented hedgehog signaling, features also seen in other PKD models. We saw increased *Gli1* (hedgehog readout) in postnatal *Six2creFrs2αKO* interstitium and ectopic sonic hedgehog (Shh) in subsets of non-dilated P7 mutant proximal tubules (likely driving the stromal Gli expression). As ectopic tubular Shh and Ccl2 expression is seen after acute kidney injury (AKI), we interrogated another bone fide AKI marker, Kim1 and noted ectopic expression in P7 non-dilated proximal tubules. These observations suggest that aberrantly activated “AKI” pathways may drive pathogenesis in PKD.

Fgfr signaling is mediated by docking proteins, including fibroblast growth factor receptor substrate 2*α* (Frs2*α*)^1^. Upon Fgf ligand binding and receptor dimerization, Frs2*α* becomes phosphorylated, leading to activation of Erk, Akt, and alternative forms of protein kinase C^1^. While global deletion of *Fgfr1*, *Fgfr2* or *Frs2α* in mice is early embryonic lethal, many investigators have shown critical roles for this pathway in renal development using ligand and/or conditional receptor knockouts^1^,^2^,^3^,^4^,^5^,^6^,^7^,^8^,^9^. Recently, our laboratory generated an allelic series of Six2cre-mediated conditional knockout mice that remove *Fgfr(s)* and/or *Frs2α* expression and function specifically in the nephron progenitor population^10^. Nephron progenitors, expressing the transcription factor Six2, give rise to glomeruli and all nephron tubular segments including proximal convoluted tubules. *Six2cre-*mediated deletion of both *Fgfr1* and *Fgfr2* in mice resulted in early depletion of nephron progenitors due primarily to excessive apoptosis and loss of stemness markers such as Cited 1; these mice developed severe renal cystic dysplasia resulting in perinatal lethality. Other mutants with ablation of *Frs2α* expression/activity, including *Six2cre;Frs2α*^*fl/fl*^ (*Six2creFrs2αKO*), had a later loss of nephron progenitors at E14.5. *Frs2α*-deficient nephron progenitors were found to have lower Cited 1 expression and increased notch activity. While surviving progenitors made nephrons leading to hypoplastic kidneys by E18.5, *Six2creFrs2αKO* kidneys became very cystic postnatally leading to death by 6 weeks of age.

Ciliopathy is a key feature of renal cystogenesis^11^,^12^,^13^. In humans with, and animal models of polycystic cystic kidney disease (PKD), causative mutations have often been identified in genes encoding for proteins that associate with primary cilia and/or that are critical for ciliary assembly and/or intraflagellar transport^14^,^15^,^16^,^17^,^18^,^19^. While these ciliary-associated proteins are often predicted act in concert, the exact role of primary ciliary dysfunction remains unknown and/or controversial. Given the association of hedgehog signaling proteins with cilia, aberrant activation of this pathway is often thought to indicate ciliary dysfunction in PKD models^12^. Moreover, there are renal cystogenesis models in which causative mutations encode for proteins with no known ciliary association^20^,^21^,^22^,^23^,^24^. Thus, while cilia are a central feature of renal cystogenesis, there are likely other critical factors driving cyst formation.

Other common features of PKD models are dysregulation of β-catenin signaling and inflammation, including excessive chemokine production and macrophage infiltration^25^,^26^,^27^,^28^,^29^,^30^. Mouse models of PKD with mutations in *KIf3α* (a gene encoding for a protein necessary for ciliary formation), or *Pkd1* (leading cause of human autosomal dominant PKD) develop excessive tubular β-catenin accumulation^31^,^32^. Transgenic mice with activating mutations in β-catenin have been shown to develop a PKD phenotype^27^. Infiltrating macrophages have often been identified in PKD models and macrophage depletion often results in amelioration of cyst growth^29^. Chemokines, such as Ccl2 (major chemotactic factor for macrophages) and Tnfα (secreted by activated macrophages) have been detected in PKD models and appear to have causative roles^28^,^33^,^34^.

In this study, we sought to determine whether *Six2creFrs2αKO* mice shared features common to other PKD models. The mutants developed cysts derived from proximal tubules that were hyperproliferative and displayed interstitial fibrosis, similar to other models. Mutant kidneys also had upregulated Wnt/β-catenin expression, macrophage and chemokine production and excessive hedgehog activity, like other models. Given the latter and that Fgf signaling in other contexts regulates cilia formation^35^ we were surprised that ciliary formation appeared intact. Moreover, we noted that subsets of non-dilated proximal tubules in young postnatal mutants ectopically expressed Shh, Ccl2, and Kim1, all known to be activated by acute kidney injury. Thus, aberrantly activated “AKI” pathways may drive renal pathogenesis in PKD.

## Results

### Cystogenesis in *Six2creFrs2αKO* kidneys

**Figure 1:**
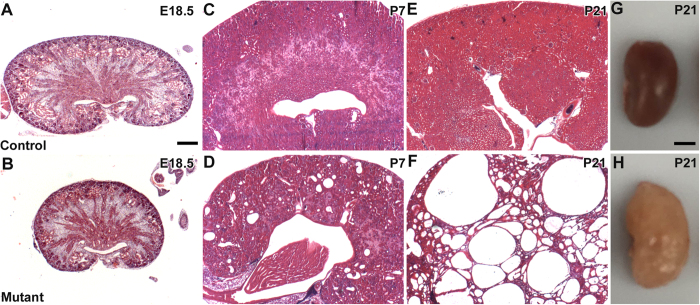
Tissue sections and gross images show progressive cyst formation in *Six2creFrs2αKO* kidneys. (**A**–**F**,**H**,**E)** Stained sections of control (**A**,**C**,**E**) and *Six2creFRS2αKO* (**B**,**D**,**F**) kidneys show that the mutants are hypoplastic at E18.5 (**A** vs. **B**), develop small cysts dispersed in normal appearing parenchyma at P7 (**C** vs. **D**), and have cysts replacing most of the parenchyma by P21 (**E** vs. **F**). (**G**,**H**). Dissected kidneys from P21 control (**G**) and *Six2creFRS2αKO* (**H**) mice show that the mutant kidneys are enlarged and have multiple fluid-filled cysts. (**A**–**F**) scale bar = 200 μm; G, H scale bar = 0.25 cm.

We sought to characterize the origin and progression of cystogenesis in *Six2creFrs2αKO* kidneys. Hematoxylin and eosin stained sections reveal that E18.5 mutant kidneys are hypoplastic but non-cystic compared to controls (Fig. 1A,B). In contrast, P7 *Six2creFrs2αKO* kidneys had numerous small cysts (Fig. 1C,D), and P21 mutant parenchyma was almost completely replaced by cysts of varying size (Fig. 1E–H). Morphometric analysis of *Six2creFrs2αKO* mice showed reduction in the kidney weight (KW) and body weight (BW) with unaltered KW/BW ratio versus controls (Supplementary Fig. 1A–C). *Six2creFrs2αKO* mice showed marked upregulation in plasma blood urea nitrogen (BUN) levels in comparison to controls indicating impaired kidney function in mutants. (Supplementary Fig. 1D). Immunostaining in P7 and P21 *Six2FRSK2αKO* kidneys with LTL and DBA, proximal tubule and collecting duct markers, respectively, revealed that virtually all of the *Six2creFRS2αKO* cysts were LTL positive/proximal tubule-derived (Supplementary Fig. 2 and not shown).

### Epithelial cell proliferation, apoptosis, and interstitial fibrosis in *Six2creFrs2αKO* kidneys

**Figure 2:**
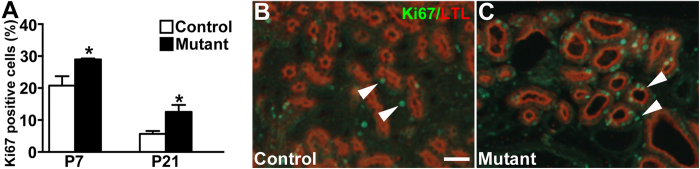
*Six2creFrs2αKO* proximal tubular-derived epithelia have increased proliferation. (**A**) Graph reveals increased percentages of Ki67 positive-nuclei in P7 and P21 mutant LTL positive tubular epithelial cells versus littermate controls (*p < 0.05). (**B**,**C**) Representative sections from a P21 control (**B**) and mutant (**C**) showing increased numbers of Ki67-positive nuclei (green, arrowheads) in LTL-positive cells (red) in the latter versus the former (DAPI staining is not shown). (**B**,**C**) scale bar = 25 μm.

**Figure 3:**
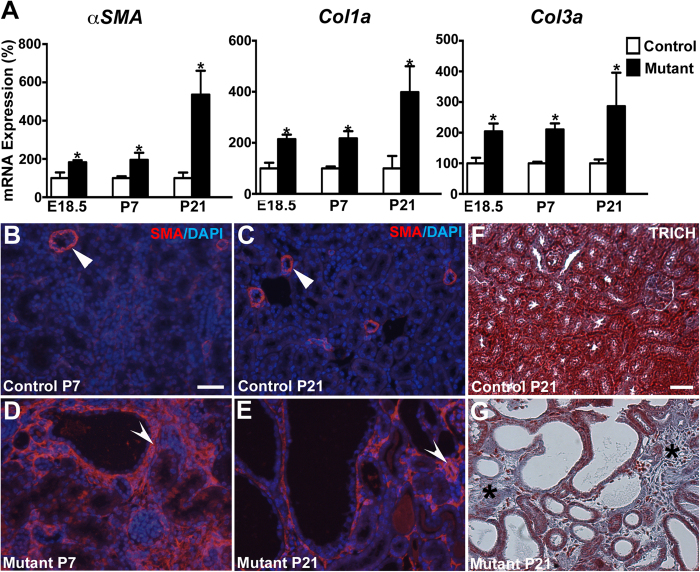
qPCR and staining reveals increased interstitial fibrosis in postnatal *Six2creFrs2αKO* kidneys. (**A**) Graphs of qPCR assays show increases in expression of fibrotic markers *αSMA*, *Col1a*, and *Col3a* in E18.5, P7 and P21 mutant kidneys versus controls. (*p < 0.05). (**B–E**) Representative immunofluorescence images for *α*SMA (SMA, red) and DAPI (blue) reveal that αSMA expression is mostly in peri-arteriolar smooth muscle in controls (arrowheads) at P7 (**B**) and P21 (**C**), whereas it is strongly expressed in the interstitium of mutants (concave arrowheads) at P7 (**D**) and P21 (**E**). (**F**,**G)** Representative Trichrome-stained sections (TRICH) shows no fibrosis in P21 controls (**F**), but significant collagen deposition in the expanded interstitium of the mutants (**G**, asterisks). (**B**–**E)** scale bar = 25 μm; (**F**,**G)** scale bar = 25 μm.

We next assessed *Six2creFrs2αKO* mice for increased epithelial cell proliferation, apoptosis, and interstitial fibrosis, other prominent features in PKD^15^,^36^,^37^,^38^,^39^,^40^. Co-staining of kidney sections with LTL, and the proliferation marker, Ki67, revealed increases in mutant proximal tubular cell proliferation rates at both P7 and P21 versus controls (Fig. 2 and not shown). TUNEL staining shows apparent increases in P21 mutant proximal tubular-derived cells vs. controls; however, the overall number of TUNEL positive cells was relatively low in both (Supplementary Fig. 3). Quantitative PCR also revealed significant increases in fibrotic markers, α*SMA*, *Col1a* (collagen 1a), and *Col3a* in E18.5, P7, and P21 mutants that worsened with age versus controls (Fig. 3A). Immunofluorescence revealed prominent αSMA staining in an expanded stromal compartment in P7 and P21 *Six2creFrs2αKO* mice versus controls, which had staining confined to peri-arteriolar smooth muscle (Fig. 3B–E). Trichrome-stained sections from P21 *Six2creFrs2αKO* kidneys showed increased collagen deposition numbers of interstitial cells versus controls (Fig. 3F,G). Together, these data show that postnatal *Six2creFrs2αKO* kidneys share histological features found in other PKD models.

### Increased and ectopic β-catenin expression in *Six2creFrs2αKO* kidneys

**Figure 4:**
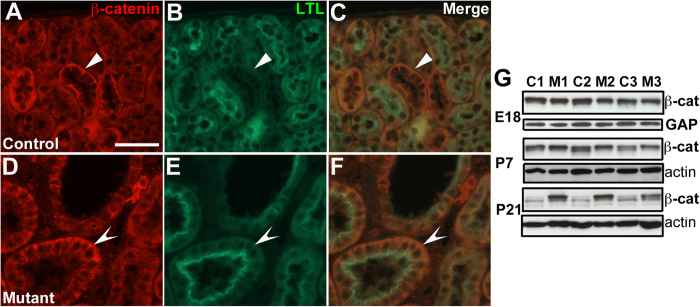
Postnatal *Six2creFrs2αKO* kidneys have ectopic and increased β-catenin expression versus controls. (**A**–**C)** Co-immunofluorescence for β-catenin (red) and LTL (green) in controls at P21 reveals β-catenin (**A**) expression in LTL-negative tubules (likely collecting ducts) (**B**) on merged images (**C**, arrowheads) (**D**–**F)**. Immunofluorescence in mutants shows ectopic β-catenin expression (**D**) in LTL-positive cysts (**E**) on merged images (**F**, concave arrowheads). (**G**) Western blots reveal equivalent β-catenin protein levels in E18.5 mutants (M1–3) and controls (C1–3), but gradually increasing levels in P7 and P21 mutants versus controls (β-cat = β-catenin, GAP = GAPDH, actin = β-actin) (cropped blots are shown as indicated by the lines- these blots were run under the same experimental conditions). (**A**–**F**) scale bar = 25 μm.

Aberrant increases in β-catenin signaling have been identified in many PKD models^25^,^26^,^32^,^41^. In our study, immunofluorescence showed that while β-catenin was only expressed in LTL-negative control tubules at P7 and P21, it was ectopically expressed in LTL-positive cysts in P7 and P21 *Six2creFrs2αKO* kidneys (Fig. 4A–F and not shown). Furthermore, while Western blots revealed equivalent β-catenin levels in E18.5 mutant and control kidneys, protein levels were marginally higher in P7 mutants and were clearly upregulated in P21 mutants versus controls (Fig. 4G). To determine whether the augmented β-catenin expression was likely driving canonical Wnt signaling, we performed qPCR for the canonical Wnt target genes *Axin2* and *Lef1* in P7 whole kidneys. Indeed, we observed increased Axin2 and a trend for increased Lef1 mRNA in mutant kidneys versus controls (Supplementary Fig. 4D). Quantitative PCR for Wnt ligands, *Wnt4*, *Wnt7b* and *Wnt9b* revealed correlative increases in mutant mRNA levels starting at E18.5 and continuing through P21 versus controls (Supplementary Fig. 4A). *In situ* hybridization revealed patches of ectopic *Wnt4* expression in P21 mutant cortical tubules/cysts and interstitium versus controls (Supplementary Fig. 4B,C). Together, these data show *Six2creFrs2αKO* kidneys have aberrant upregulation of Wnt ligands and canonical β-catenin signaling.

### Macrophage infiltration and inflammatory genes in *Six2creFrs2αKO* kidneys

**Figure 5:**
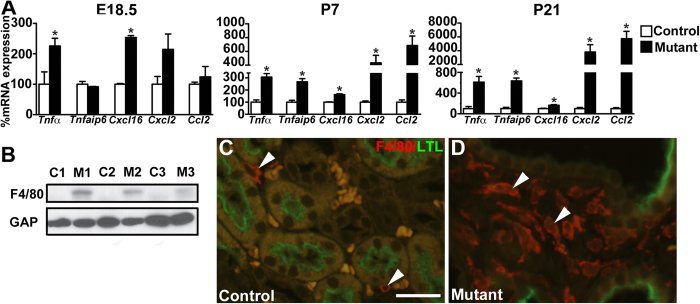
*Six2creFrs2αKO* kidneys have increases in inflammatory cytokines and macrophage infiltration. (**A**) Graphs of qPCR assays from whole kidneys shows up-regulation in mutant *TNFα*, *Tnfaip6*, *Cxcl16*, *Cxcl2*, and *Ccl2* mRNA versus controls, that worsens with age (note *Cxcl2* and *Ccl2* relative expression levels at P21) (*p < 0.05). (**B**) Western blot reveals that P21 mutants (M1-3) have increased F4/80 protein expression than controls (C1-3) (cropped blots are shown as indicated by the lines- these blots were run under the same experimental conditions). (**C**,**D)** Co-immunofluorescence for F4/80 (red) and LTL (green) shows that while P21 controls have few red-staining macrophages in the interstitium (**C**, arrowheads), mutants have many macrophages in the interstitium (**D**, arrowheads); note that the orange-staining cells in the control image are red blood cells. (GAP = GAPDH). Scale bar = 25 μm.

We next examined whether *Six2creFrs2αKO* kidneys had increased expression of inflammatory cytokines, particularly those that recruit monocytes/macrophages (e.g. Ccl2) or that are secreted by activated macrophages (e.g. Tnfα and Cxcl2), as in other PKD models^28^,^29^,^30^,^33^,^40^,^42^. From E18.5 to P21, we noted increasing mutant kidney *Tnfα*, *Tnfaip6* (a Tnfα readout), *Cxcl16*, *Cxcl2*, and *Ccl2* expression versus controls by qPCR (Fig. 5A). The most striking increases were in P21 mutant *Cxcl2* and *Ccl2* levels. We then examined *Six2creFrs2αKO* kidneys for macrophage infiltration, which is also seen in other PKD models^29^. We observed increased F4/80 protein expression in P21 mutant kidneys by Western blot (Fig. 5B) and many more F4/80-positive macrophages by immunofluorescence in P7 and P21 mutant interstitium (Fig. 5C,D and not shown) than controls.

**Figure 6:**
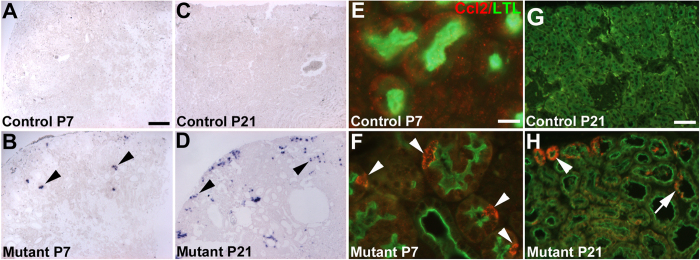
Ccl2 is ectopically expressed initially in non-dilated *Six2creFrs2αKO* proximal tubular cells. (**A**,**B**) At P7, *in situ* hybridization reveals no apparent *Ccl2* expression in controls (**A**), but diffuse punctate staining exclusively in non-dilated cortical tubular cells in mutants (**B**, arrowheads). (**C**,**D**) At P21, *in situ* hybridization again shows no *Ccl2* staining in controls (**C**), but reveals a larger number of mostly non-dilated mutant cortical cells with ectopic expression of Ccl2 (**D**, arrowheads). (**E**,**F)** At P7, co-immunofluorescence for LTL (green) and Ccl2 (red) reveals no Ccl2 expression in controls (**E**), but ectopic Ccl2 expression in individual cells within non-dilated LTL-positive proximal tubules in mutants (**F**, arrowheads). (**G**,**H)** At P21, co-labeling immunofluorescence reveals no signal in controls (**G**), but strong labeling of minimally-dilated LTL-positive tubules in the outer cortex (arrowhead) and of individual cells within LTL-positive cyst lining cells (arrow). (**A**–**D**) scale bar = 100 μm; (**E**,**F**) scale bar = 12.5 μm; (**G**,**H**) scale bar = 25 μm.

We then sought to determine the source of the increased mutant Ccl2 expression (that was likely driving macrophage infiltration). In controls, we detected no obvious *Ccl2* expression by *in situ* hybridization; however, P7 mutants had punctate ectopic staining exclusively in non-dilated renal cortical epithelial cells. P21 mutants had more widespread *Ccl2* expression, mostly in minimally dilated tubules, but occasionally in cyst lining cells (Fig. 6A–D). Dual-label immuno-fluorescence for Ccl2 and LTL revealed no Ccl2 protein expression in controls at any age or mutant kidneys at E18.5 (Fig. 6E,G, Supplementary Fig. 8E,F); however, at P7 individual Ccl2-positive cells were present within non-cystic LTL-positive mutant proximal tubules (Fig. 6F). At P21, Ccl2 protein was ectopically expressed most robustly in minimally-dilated mutant proximal tubules, and to a lesser degree in LTL-positive cyst-lining cells (Fig. 6H). Thus, the ectopic Ccl2 signal in *Six2creFrs2αKO* kidneys emanates from non-dilated proximal tubules at P7 and from both LTL-positive non-dilated and cyst lining cells later.

### Cilia and Hedgehog signaling in *Six2creFrs2α*KO kidneys

We next determined whether cilia are present in Frs2α-deficient proximal tubular cells, given that disruption of cilia formation or function are often associated with PKD^15^,^31^,^43^. Co-staining of tubular epithelial cells isolated from P21 *Six2creFRS2αKO* and control kidneys with aquaporin-1 (proximal tubular marker) and acetylated α-tubulin (ciliary marker) revealed normal-appearing cilia in mutants versus controls (Supplementary Fig. 5A–D). Also, qPCR showed that P21 *Six2creFRS2αKO* and control kidneys have equivalent expression of several genes critical for cilia formation (Supplementary Fig. 5E).

**Figure 7:**
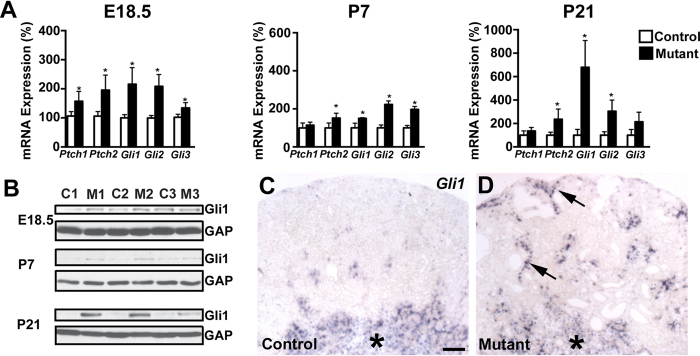
*Six2creFrs2αKO* kidneys have evidence of augmented hedgehog signaling. (**A**) Graphs of quantitative PCR analyses from control and mutant kidneys reveal relative age-dependent increases in mRNA of hedgehog signaling components, *Ptch1*, *Ptch2*, *Gli1*, *Gli2*, and *Gli3*. (**B)** Western blot analysis reveals increases in mutant (M1–3) kidney expression of Gli1 protein, a strong readout of hedgehog activity, starting at E18.5, but increasing at P7 and P21 versus controls (C1–3) (cropped blots are shown as indicated by the lines- these blots were run under the same experimental conditions). (**C**,**D**) *in situ* hybridization at P7 reveals similar medullary *Gli1* expression in controls (**C**, asterisk) and mutants (**D**, asterisk), but ectopic expression in mutant cortical interstitium, often adjacent to cyst lining cells (**D**, arrowheads). (GAP = GAPDH). (**C**,**D**) scale bar = 100 μm.

Normal appearing cilia can be dysfunctional and augmented hedgehog signaling has been thought to indicate ciliary dysfunction in cyst lining cells in PKD models^12^. Thus, we determined whether hedgehog signaling is mis-regulated in *Six2creFRS2αKO* cystic kidneys. Quantitative PCR revealed increases (or trends for increases) in mutants from E18.5 to P21 for multiple readouts of hedgehog activity, including Patched 1–2, Gli1–3, and Smoothened mRNA (Fig. 7A). Western blots confirmed increased *Six2creFrs2αKO* kidney Gli1 expression, a strong indicator of hedgehog signaling versus controls, increasing from E18.5 to P21 (Fig. 7B). To determine the target cells of the increased hedgehog activity in the mutant kidneys, we examined *Gli1* expression by *in situ* hybridization. In P7 controls and mutants, we observed similar medullary *Gli1* staining; however, mutants had ectopic *Gli1* expression in the renal cortical interstitium, usually adjacent to cysts, but almost never in cyst lining cells (Fig. 7C,D). At P21 and P28, we again found that virtually all of the ectopic mutant *Gli1* expression was in the expanded interstitium near cysts, and excluded from the cyst lining cells (Supplementary Fig. 6 and not shown). Thus, the primary target of the mutant ectopic hedgehog signaling appears to be interstitial cells and not cyst lining cells, suggesting that the augmented hedgehog activity is not likely a readout of cyst lining cell ciliary dysfunction.

**Figure 8:**
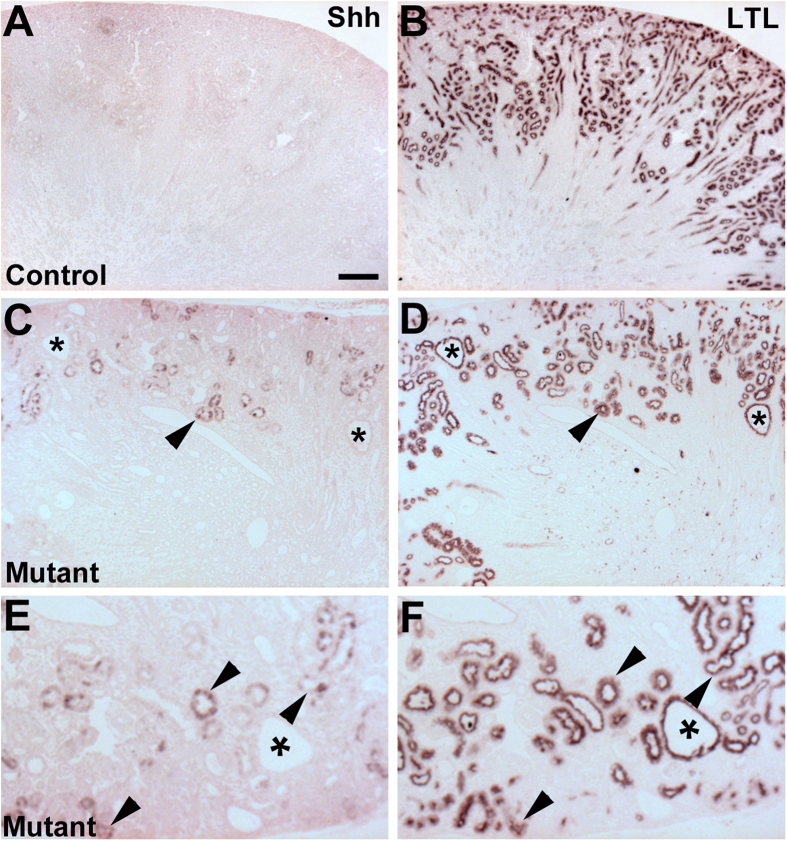
P7 *Six2creFrs2αKO* kidneys have ectopic Shh staining in non-dilated proximal tubules. (**A**,**B**) Immunostaining for Shh and LTL in adjacent P7 control kidneys (A and B, respectively) reveals no obvious Shh expression (**A**). (**C**,**D)** Immunostaining in P7 mutant kidneys shows clusters of ectopic Shh staining in non-dilated tubules (**C**, arrowhead) that also express LTL in an adjacent section (**D**, arrowhead), but shows no Shh staining in LTL-positive cyst lining cells (asterisks). (**E**,**F**) Higher magnification from regions in panels (**C**,**D)** (respectively), confirm that non-dilated Shh-expressing mutant tubules are also LTL-positive (arrowheads), but that LTL-positive cysts do not express Shh (asterisks). (**A**–D) scale bar = 100 μm.

We then sought to determine the source driving the increased *Six2creFrs2αKO* kidney hedgehog signaling. Quantitative PCR analysis from mRNA isolated from P7 mutant and control kidneys showed a significant increase Sonic Hedgehog transcript levels in the mutant vs. control whereas, Indian and Desert Hedgehog mRNA levels were not significantly different (Supplementary Fig. 7A). Furthermore, immunostaining for Shh and LTL in adjacent sections at P7 revealed no Shh expression in control cortex (Fig. 8A), but many mutant cortical tubules with ectopic staining that were also all LTL-positive (Fig. 8C–F). Moreover, the ectopic staining was only noted in non-dilated proximal tubules and excluded from cyst lining cells. Notably, we could not detect any ectopic increase in Shh expression in E18.5 mutant kidneys by immunostaining, making this something that occurs in postnatal mature tubules (Supplementary Fig. 8A,B). At P21, we also did not observe any further ectopic Shh staining in *Six2creFrs2αKO* kidneys. Thus, it appears that ectopic mutant Shh protein in non-dilated proximal tubules in early postnatal kidneys likely triggers the ectopic *Gli1* expression in the mutant interstitium (and that the cyst lining cells are not driving the augmented hedgehog response).

### *Six2creFrs2αKO* proximal tubular-derived cells have upregulation of the acute kidney injury marker, Kim1

**Figure 9:**
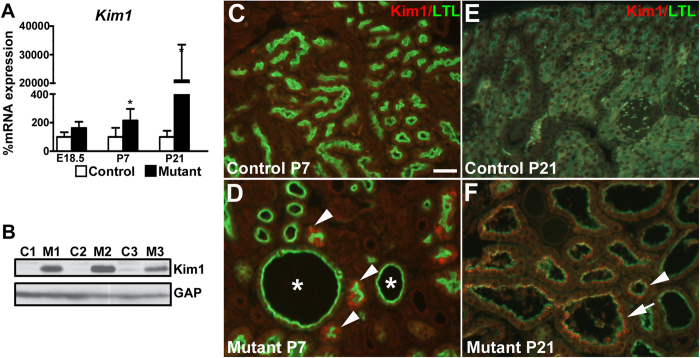
Kim1 is ectopically expressed initially in *Six2creFrs2αKO* non-dilated proximal tubular cells. (**A)** Quantitative PCR analysis reveals increases in mutant kidney *Kim1* mRNA expression versus controls starting at P7 and worsening at P21. (**B**) Western blot analysis at P21 confirms a significant increase in Kim1 protein in mutant kidneys (M1-3) versus controls (C1–3) (cropped blots are shown as indicated by the lines- these blots were run under the same experimental conditions). (**C**,**D**) Co-immunofluorescence for Kim1 (red) and LTL (green) at P7 reveals that while controls display no Kim1 expression (**C**), subsets of mutant non-dilated LTL-positive proximal cells ectopically express Kim1 (**D**, arrowheads), whereas mutant LTL-positive cysts (**D**, asterisks) do not. (**E**,**F**) Co-immunofluorescence for Kim1 (red) and LTL (green) at P21 again shows no obvious Kim1 expression in control kidneys (**E**), whereas many minimally-dilated and overtly cystic LTL-positive mutant cells ectopically express Kim1 (F, arrowhead and arrow, respectively). (**A**,**B)** scale bar = (**C**–**F**) scale bar = 25 μm.

Given that ectopic proximal tubular Shh and Ccl2 expression has been observed in models of acute kidney injury^44^,^45^,^46^, we assessed whether *Six2creFrs2αKO* kidneys had ectopic expression of another bone fide marker of acute kidney injury, Kim1^47^,^48^. Quantitative PCR from E18.5 to P21, revealed significantly elevated levels of mutant *Havcr1* (the gene that encodes Kim1) versus controls by P7 and that were exaggerated by P21 (Fig. 9A). Western blot confirmed marked increases in P21 mutant kidney Kim1 levels versus controls. Although Kim1 expression was undetectable in control and mutant kidneys at E18.5 (Supplementary Fig. 8C,D), co-immunofluorescence in P7 mutants revealed patches of non-dilated LTL-positive/Kim1-positive cells, often near cysts, but excluded from the cyst lining cells, versus no staining in controls (Fig. 9C,D). By P21, we detected ectopic Kim1 staining in many LTL-positive mutant cells, including those present in minimally dilated tubules and in overt cysts (Fig. 9E,F). Together, it appears that many of the “normal” appearing non-dilated *Six2creFrs2αKO* proximal tubules at P7 have ectopic expression of acute kidney injury markers, some of which persist in the LTL-positive cyst lining cells at P21.

## Discussion

We found that kidneys lacking Frs2*α* in nephron progenitors develop rapid cyst growth after birth. The mutant kidneys show increased proliferation of proximal tubular cells, progressive interstitial fibrosis, and macrophage infiltration, consistent with other PKD models. While *Six2creFrs2αKO* kidney weight/body weight ratios were not increased versus controls (as happens with other PKD models), we suspect that this is due the fact that the mutants have perinatal renal hypoplasia due to the previously reported E14.5 loss of nephron progenitors^10^ (and thus make fewer nephrons that even when becoming cystic do not result in massively enlarged kidneys). There are no reports of nephron progenitor depletion in other PKD models, which makes this finding unique to this model. While there appears to also be an increase in apoptosis in postnatal mutant proximal tubular cells, the overall rates in both mutants and controls appear low, making excess apoptosis only a minor contributor to the mutants not having significantly enlarged kidneys. Older *Six2creFrs2αKO* mice do have signs of advanced kidney failure with the elevated BUN levels versus littermate controls, consistent with what happens at advance stages of PKD. At the molecular level, *Six2creFrs2αKO* kidneys show aberrant upregulation of many signaling components, including β-catenin, inflammatory chemokines (e.g. Tnfα, Ccl2), and hedgehog, all of which are observed in other PKD models^15^,^28^,^32^,^33^,^42^. A novel finding in our model is that at early stages, we observe increases in tubular acute kidney injury markers (Shh, Ccl2, and Kim1), which likely has a significant role in driving disease pathogenesis.

Dysregulated β-catenin signaling, excessive chemokine production and macrophage infiltration are often seen in PKD models^25^,^26^,^31^,^32^,^41^. Excessive β-catenin accumulation has been observed in cystic models, including *Pkd1* and *Kif3α* mutants, and appears to drive cystogenesis^25^,^26^,^27^,^31^,^32^,^41^. Thus, is it likely that ectopic β-catenin expression in *Six2creFrs2αKO* kidneys, which appears to be stimulated by increased Wnt ligand expression and appears to be a readout of canonical Wnt signaling, contributes to cyst growth. Excessive production of inflammatory chemokines that recruit macrophages (e.g. Ccl2) and that are secreted by activated macrophages (e.g. Tnfα) have been identified in and appear to have a causative role in many PKD models^28^,^33^,^42^. As in these models, *Six2creFrs2αKO* mice have excessive chemokine production and macrophage infiltration, which likely also drive cystogenesis. Thus, *Six2creFrs2αKO* mice share many features seen in other bone fide PKD models, and offer a novel model to interrogate mechanisms driving cystogenesis.

Another molecular pathway perturbed in *Six2creFrs2αKO* mice and in other PKD models is hedgehog. The qPCR profile in *Six2creFrs2αKO* kidneys revealed increases in hedgehog readouts (Fig. 7A) and has a marked resemblance to the profile in conditional knockouts of *Pkd1* and *Thm1* (a ciliary associated gene), established PKD models^15^. Furthermore, *Thm1* mutants with concomitant deletion of *Gli2* have diminished cystogenesis, indicating that increased hedgehog signaling contributes towards cyst progression in that model^15^. How aberrant hedgehog signaling contributes to cystogenesis in PKD remains to be defined.

Alterations in hedgehog signaling in PKD models are often thought to reflect ciliary dysfunction^12^. In *Six2creFrs2αKO* mice, however, ciliary structure in affected proximal tubular epithelial cells appeared normal, based on histology and in the qPCR profile for genes critical for ciliary formation^12^,^15^. While normal ciliary structure does not mean normal function, the ectopic *Gli1* expression in *Six2creFrs2αKO* was most notable in the expanding interstitium and was rarely observed in cyst lining cells. This makes it unlikely that aberrant hedgehog signaling is a readout of ciliary dysfunction in our model (and perhaps in other PKD models).

Another question not often addressed in most PKD models is what drives the ectopic Gli1 expression. *Six2creFrs2αKO* kidneys have what appears to be ectopic Shh protein in subsets of non-dilated proximal tubular cells at P7 (and again, not in cystic epithelium). The protein expression most likely reflects Shh as opposed to Ihh or Dhh, given that qPCR only shows increases in mutant *Shh* transcripts (and not the others) versus controls at P7. The proximity of the ectopic Shh expression to the ectopic *Gli1* in the interstitium makes it likely that the former drives the latter. Moreover, many data show that enhanced renal interstitial Gli1 activity leads to fibrosis^44^,^49^,^50^, making it likely that the ectopic hedgehog in *Six2creFrs2αKO* kidneys contributes to interstitial fibrosis. Perhaps ectopic Shh expression in non-dilated tubules has not been appreciated in other models (e.g. homozygous *Pkd1* or *Pkd2* mutants) due to early and rapid cyst progression making it difficult to assess non-dilated tubules.

Given that ectopic tubular Shh and Ccl2 expression has been noted after acute kidney injury^44^,^45^,^46^, we interrogated Kim1, a bone fide marker of AKI^47^,^48^, and noted ectopic expression early in subsets of *Six2creFrs2αKO* proximal tubules. While ectopic Kim1 production has been noted in other PKD models, its expression in non-cystic renal epithelium has not to our knowledge been documented^48^. Moreover, Kim1 can drive Ccl2 expression and thus may play an active role in driving pathogenesis in our and other PKD models^51^. Perhaps *Six2creFrs2αKO* tubules (and tubules in other PKD models) are “sensitized” to otherwise non-injurious stimuli, leading to the ectopic production of the aforementioned proteins. Moreover, the potential for overt AKI having a deleterious role in driving PKD pathogenesis is supported by studies showing that ischemic AKI superimposed on postnatal mice with induced *Itf88* or *Kif3α* homozygous deletion (leading to deciliation) or with a one-allele loss of *Pkd1* accelerates cyst progression compared to non-ischemic mice^36^,^52^,^53^. In mouse models and in human forms of dominant PKD, there has long been an assertion that a “second hit” is required for cyst growth that often starts later in life^54^. Perhaps subclinical injuries in sensitized tubules and/or overt AKI may often be that second hit.

## Methods

### Mice

*Frs2α*^*fl/fl*^ and transgenic *Six2creEGFP* mice^55^,^56^, were bred to generate *Six2creEGFP;Frs2α*^*fl/fl*^ mice (*Six2creFrs2αKO*) mice as described^10^. Cre negative littermates were used as controls. All experiments were carried out with the approval of the University of Pittsburgh Institutional Animal Care and Use Committee in accordance with the guidelines of the Association for Assessment and Accreditation of Laboratory Animal Care.

### Whole mouse, kidney and blood urea nitrogen (BUN) measurements

P29 *Six2creFrs2αKO* and control mice (n = 4) were subjected to general anesthesia and blood removed by ventricular puncture leading to euthanasia. The mice were weighed after which kidneys were dissected and weighed. Blood was placed in heparinized tubes and plasma was obtained after centrifugation at 2000 rcf for 15 min. Plasma was sent to the Kansas State Veterinary Diagnostic Laboratory, which performed BUN levels.

### Western blot

Briefly, as described^57^,^58^,^59^, whole cell lysates were prepared from kidneys isolated from E18.5, P7 and P21 control and mutant mice and homogenized in RIPA lysis buffer (Thermo Scientific, MA, USA) with a protease inhibitor cocktail, sonicated for 10s and rocked for 15 min at 4 °C and then pelleted at 12,000 × g for 15 min. Supernatant was mixed in 2X Laemmli sample buffer (Bio-Rad #161–0737). Whole-cell extracts were separated on a 10% SDS-PAGE gel and resolved proteins were electrophoretically transferred to nitrocellulose membranes, which were then washed with Tris-buffered saline (TBS). Membranes were blocked and probed with primary antibodies (at dilutions listed in Supplementary Table 1) followed by incubation with horseradish peroxidase-conjugated rabbit or mouse second antibodies. The antigen-antibody complex was visualized with Amersham^TM^ ECL^TM^ prime Western blotting detection reagent. (GE Healthcare UK Limited Buckinghamshire UK, # RPN2232).

### Immunofluorescence

Briefly, as described^2^,^57^, 4 μm paraformaldehyde (PFA)-fixed paraffin embedded kidney sections were deparaffinized, rehydrated and subjected to antigen retrieval in citrate buffer (10 mm citrate, 0.1% Tween-20, pH 6.0) at 95 °C for 30 min and then kept at room temperature for 30 min. The sections were blocked for 1 h in donkey serum at room temperature and incubated for 12–24 h with primary antisera (see Supplemental Table 1). Alexa Fluor 594 and Alexa Fluor 488 secondary antibodies were used and nuclei were stained with 4′6′-diamidino-2-phenylindole (DAPI). Staining was detected using a Leica DM 2500 fluorescence microscope.

### Cell proliferation and apoptosis

To determine proliferation rates of LTL positive cells, cross sections through the midpoint of mutant and control kidneys (n = 3 per genotype) were co-stained with the proliferation marker Ki67 and LTL. Ki67/LTL double positive cells and DAPI/LTL double positive cells were counted and proliferation rates were determined by dividing the former by the latter. To assay for apoptosis, Terminal deoxynucleotidyl transferase-mediated UTP end labeling (TUNEL) was performed with ApopTag Plus *In Situ* Apoptosis Fluorescein Detection kit (EMD Millipore S7111-Kit) according to the manufacturer’s protocol. To identify LTL-positive cells, the sections were incubated with biotinylated LTL followed by staining with Alexa Flour 594 streptavidin.

### Proximal tubule-enriched primary cells

Briefly, as described^60^, P21 kidney cortices were digested with collagenase for 20 min at 37 °C and passed sequentially through 180 μm, 90 μm and 40 μm diameter sieves. Tubules between 40 and 90 μm were plated on fibronectin coated coverslips in DMEM/F12 medium with insulin, transferrin, selenite, hydrocortisone, Egf, triiodothyronine and penicillin-streptomycin for 7 days. Cells were then fixed in 4% paraformaldehyde, permeabilized, and immunostained as above (see Supplemental Table 1 for primary antibodies).

### *In situ* hybridization

Briefly, as described^6^,^10^,^58^, 7 μm PFA-fixed, paraffin-embedded tissue sections were deparaffinized, rehydrated and treated with proteinase K (10 *μ*g/ml) for 30 minutes, re-fixed in PFA, incubated in 0.1% HCl for 10 min followed by rocking in 0.14 M triethanolamine and 0.25% acetic anhydride for 10 min. Slides were washed and blocked with hybridization buffer and incubated with digoxigenin (DIG)-labeled riboprobe for 12–15 h at 68 °C. Slides were washed, treated with RNase, blocked, and incubated with anti–DIG-AP antibody (Roche, Indianapolis, IN) overnight. Slides were then washed and developed with chromogenic substrate (BM Purple; Roche) for a maximum of 3 days, fixed and mounted with Fluoromount-G (Southern Biotech, Birmingham. Al). Probe templates were generated by *in vitro* transcription from plasmids containing bases 34–587 of *Wnt4* (NM009523.2) and 48–556 of *Ccl2* (NM011333) coding regions, respectively. The *Gli1 template was gifted by Dr. Alexandra Joyner*^61^. Digoxigenin labeled antisense probes were generated with DIG labeling kits (Roche).

### Quantitative real time PCR

RNA was extracted from snap frozen kidneys (n = 3 for E18.5 and P21; n = 4 for P7 samples) using the RNAeasy Plus MicroKit (Qiagen, Valencia, CA). Primer sequences are listed in Supplemental Table 2. The Superscript First Strand cDNA kit (Invitrogen, Waltham, MA) and a C1000 Thermal Cycler (Bio-Rad, Hercules, CA) were used to perform the qPCR assays. Reactions were conducted in a total volume of 12.5 μL containing 2.5 μL of cDNA, 6.25 μL of 2X Sso advanced SYBR Green Supermix (Bio-Rad, #1725261), and each primer at a concentration of 0.1 μM. Primer pairs were independently validated for use by melt curve analysis and gel electrophoresis to confirm the amplification of a single PCR product of expected size. For negative controls, a qPCR reaction was performed using cDNA obtained from reverse transcription reactions lacking reverse transcriptase. The qPCR analysis was initiated by melting of cDNA at 95 °C for 3 min and followed by 40 amplification cycles of 30 sec at 95 °C and 1 min at 60 °C. Dissociation-curve analysis was performed and Ct values were recorded and analyzed via the ΔΔCt method to characterize relative fold-changes in mRNA expression between treatment groups. *Gapdh* was used for normalization.

### Statistical analyses

Statistical analyses of qPCR experiments were performed on biological replicates using a Student’s *t*-test. Values are represented as means ± standard error with p < 0.05 considered significant.

## Additional Information

**How to cite this article**: Puri, P. *et al.* *Six2creFrs2α* knockout mice are a novel model of renal cystogenesis. *Sci. Rep.* **6**, 36736; doi: 10.1038/srep36736 (2016).

**Publisher’s note**: Springer Nature remains neutral with regard to jurisdictional claims in published maps and institutional affiliations.

## Supplementary Material

Supplementary Information
