# Supplementary material for: Six2creFrs2α knockout mice are a novel model of renal cystogenesis
Source: Sci Rep. 2016 Nov 17;6:36736. doi: 10.1038/srep36736 (PMC5113122; doi:10.1038/srep36736)
Supplement: Supplementary Information [file srep36736-s1.pdf]

Supplementary Information:

Six2creFrs2α knockout mice are a novel model of renal cystogenesis

Pawan Puri, PhD[1], Daniel Bushnell, BS[1], Caitlin M. Schaefer, BA[1], Carlton M. Bates, MD[1,2]

[1]Division of Nephrology, Department of Pediatrics, University of Pittsburgh School of Medicine, Pittsburgh, PA.

[2]Children's Hospital of Pittsburgh of UPMC, Pittsburgh, PA

Corresponding author:

Carlton M Bates

Children's Hospital of Pittsburgh of UPMC

University of Pittsburgh School of Medicine

4401 Penn Avenue

Pittsburgh, PA 15224

Email: batescm@upmc.edu

Phone: 412-692-9440

Fax: 412-692-7756

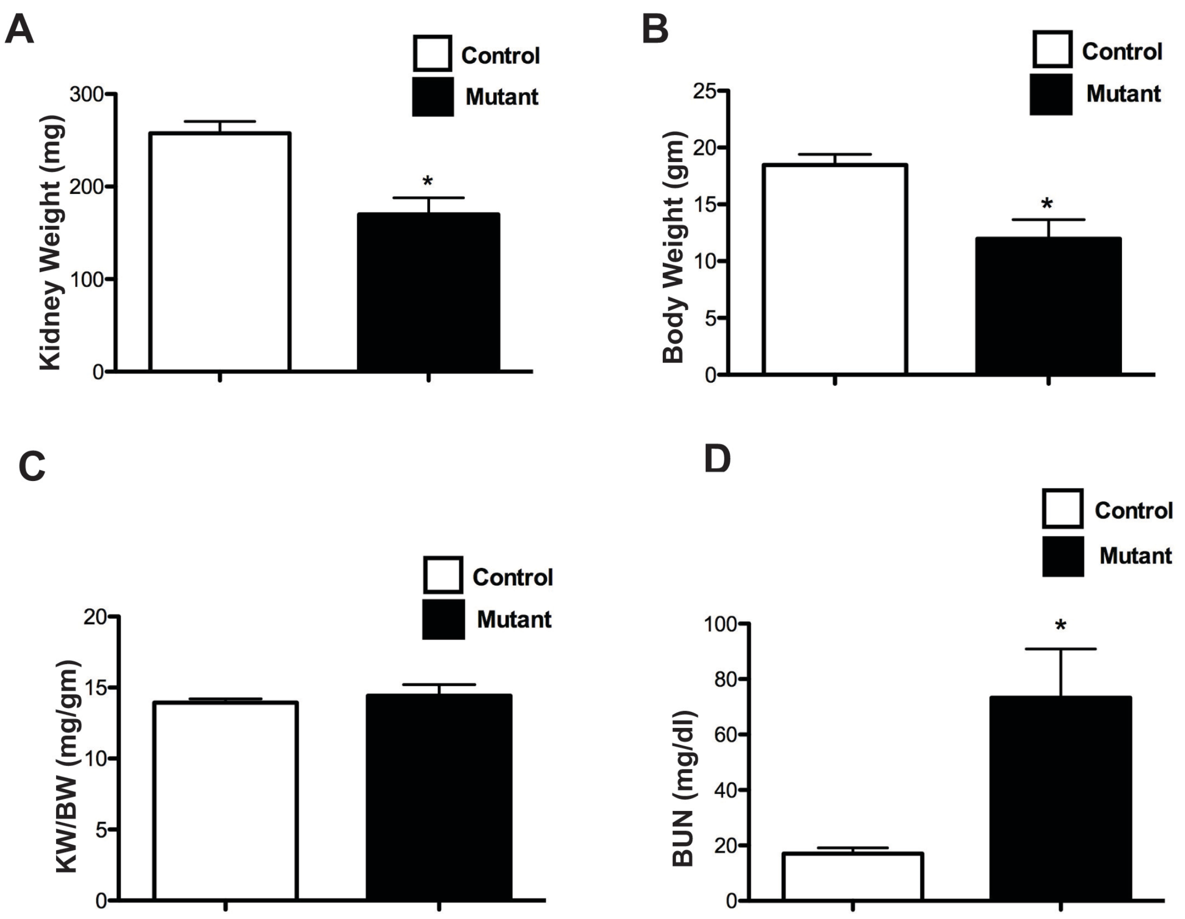

Supplementary Figure 1: Body weight, kidney weight and renal function in P29 *Six2Frs2αKO* mice.

A-C. Absolute mean kidney weight (KW) (A) and body weight (BW) (B) of P29 *Six2Frs2αKO* mice are significantly reduced versus controls whereas, the KW/BW ratio is not altered. Plasma blood urea nitrogen levels (BUN) are higher in mutants than control. Bars represent mean ± SEM of 4 control and 4 mutant mice (*$p<0.05$).

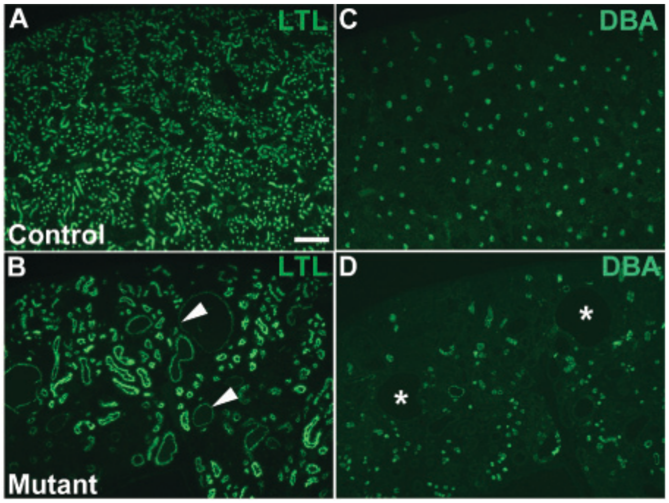

Supplementary Figure 2: Immunofluorescence in P7 *Six2creFrs2αKO* kidneys shows that cyst lining cell originate from proximal tubules.

A, B. Immunofluorescence with LTL lectin (green) labels normal proximal tubules in controls (A) and both non-dilated tubules and cysts in the mutants (B, arrowheads). C, D. Immunofluorescence with DBA lectin (green) labels normal-appearing collecting ducts in controls (C) and mutants (D) and does not label mutant cyst lining cells (D, asterisks). A-D scale bar = 100 μm.

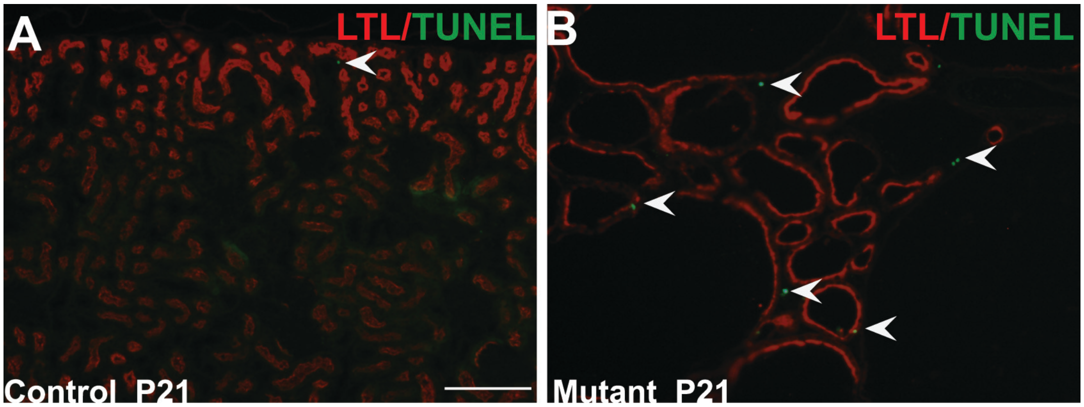

Supplementary Figure 3: Representative TUNEL assay images in P21 control and *Six2creFRS2αKO* kidneys reveal marginal increase in apoptosis in mutant kidneys.

Co-immunostaining of P21 control (A) and *Six2creFrs2αKO* kidneys (B) with TUNEL (green, arrowheads) and LTL (Red) shows an apparent marginal increase in the number of apoptotic proximal tubular derived cells in the mutant kidney. Scale bar = 100 μm.

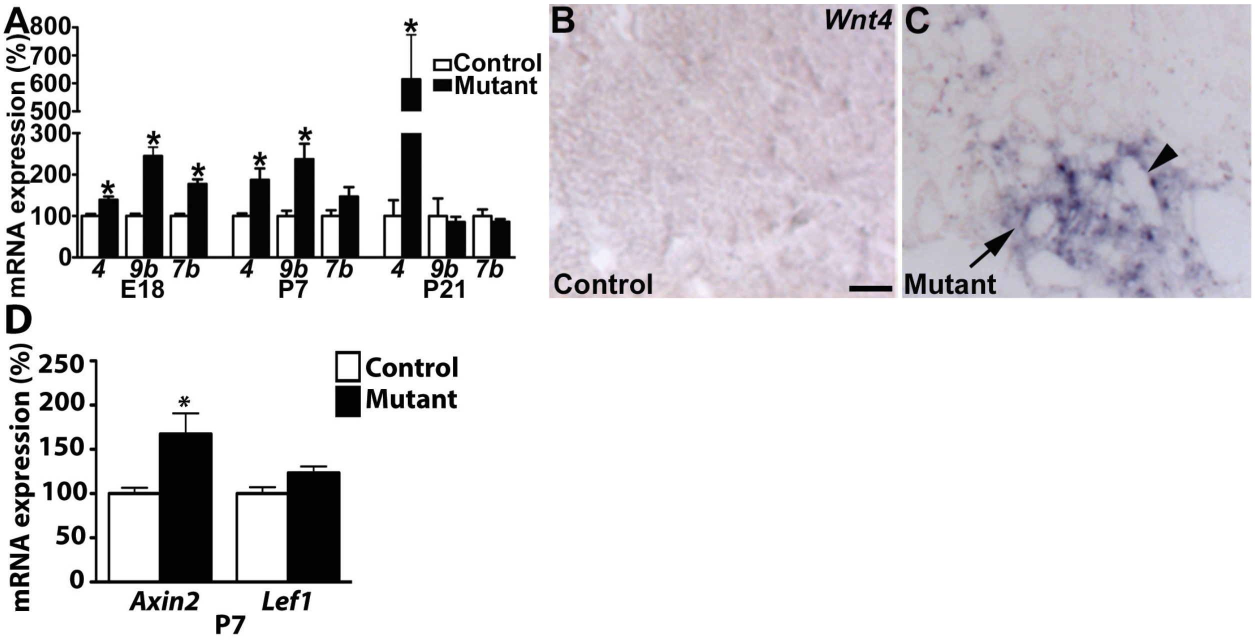

Supplementary Figure 4: Wnt ligand expression and canonical Wnt signaling readouts are increased in *Six2creFrs2αKO* kidneys.

A. Graphs of qPCR reveal variable age-based increases in mutant *Wnt4* (*4*), *Wnt9b* (*9b*), and *Wnt7b* (*7b*) mRNA expression. B, C. In situ hybridization in P21 renal cortical regions reveals no Wnt4 expression in controls (B), but ectopic expression in mutant (C) cysts/tubules (arrowhead) and interstitium (arrow). B, C scale bar = 50 µm. (D) Graphs of qPCR assays from whole kidneys shows up-regulation of canonical Wnt target transcript *Axin2* and a trend for *Lef1* (p=0.06) in P7 mutant versus controls (n=4). (*p< 0.05).

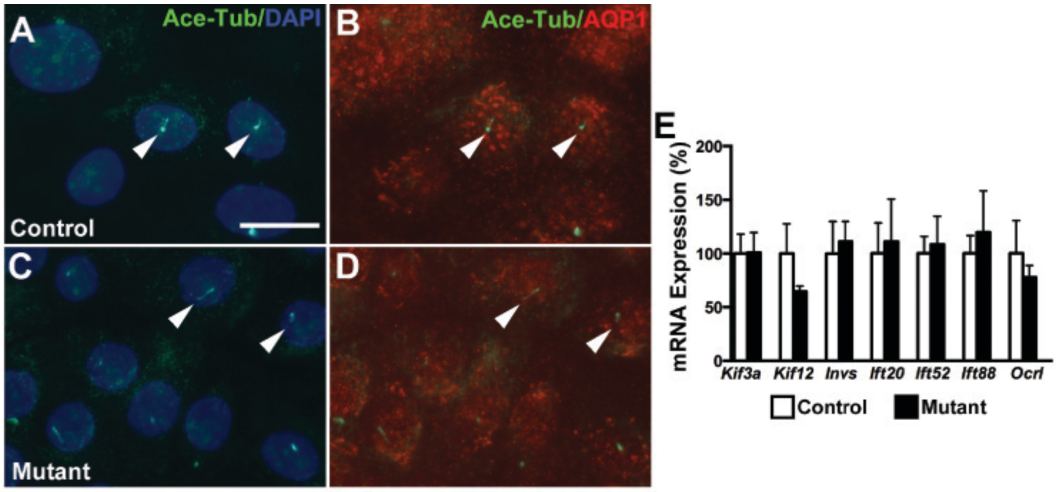

Supplementary Figure 5: *Six2creFrs2αKO* proximal tubule-derived cells appear to have normal cilia.

A-D. Co-immunofluorescence from representative P21 control (A, B) and mutant (C, D) sections reveal aquaporin-1 (AQP1) expressing proximal tubular derived cells (red) with acetylated α−tubulin (Ace-Tub) positive cilia (green, arrowheads) that appear of comparable length. E. Graphs of qPCR analyses reveal comparable expression of ciliary-associated genes *Kif3α*, *Kif12*, *Invs*, *Ift20*, *Ift52*, *Ift88*, and *Ocr1* in P21 control and mutant kidneys. DAPI = nuclear stain. A-D Scale bar = 12.5 μm.

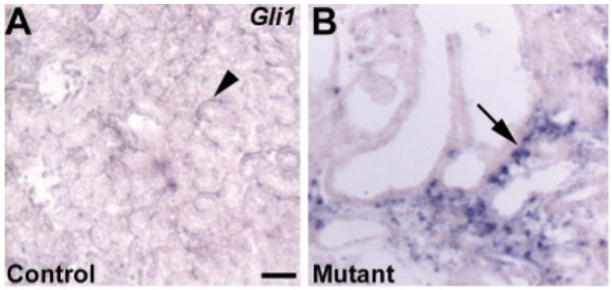

Supplementary Figure 6: P28 *Six2creFrs2αKO* kidneys display ectopic Gli1 expression in the interstitium.

A, B. in situ hybridization at P28 reveals thin linear Gli1 expression in control cortical interstitium (A, arrowhead), but strong ectopic expression in expanded mutant cortical interstitium, often adjacent to (and virtually excluded from) cyst lining cells (B, arrowhead). A-B scale bar = 25 μm.

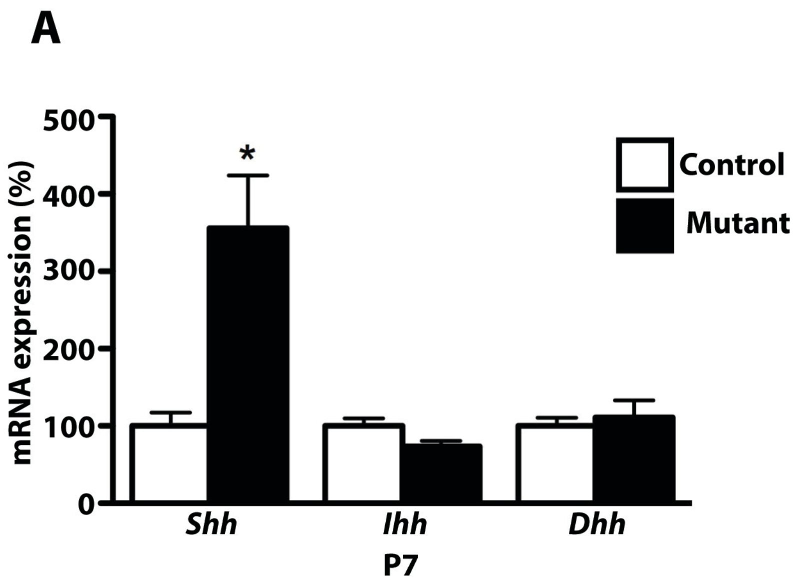

Supplementary Figure 7: Hh Ligands in P7 *Six2creFrs2αKO* kidneys

Graphs of qPCR assays from whole kidneys shows up-regulation of Shh in the mutant versus control whereas Ihh and Dhh levels are not statistically different (n=4) (*p<0.05).

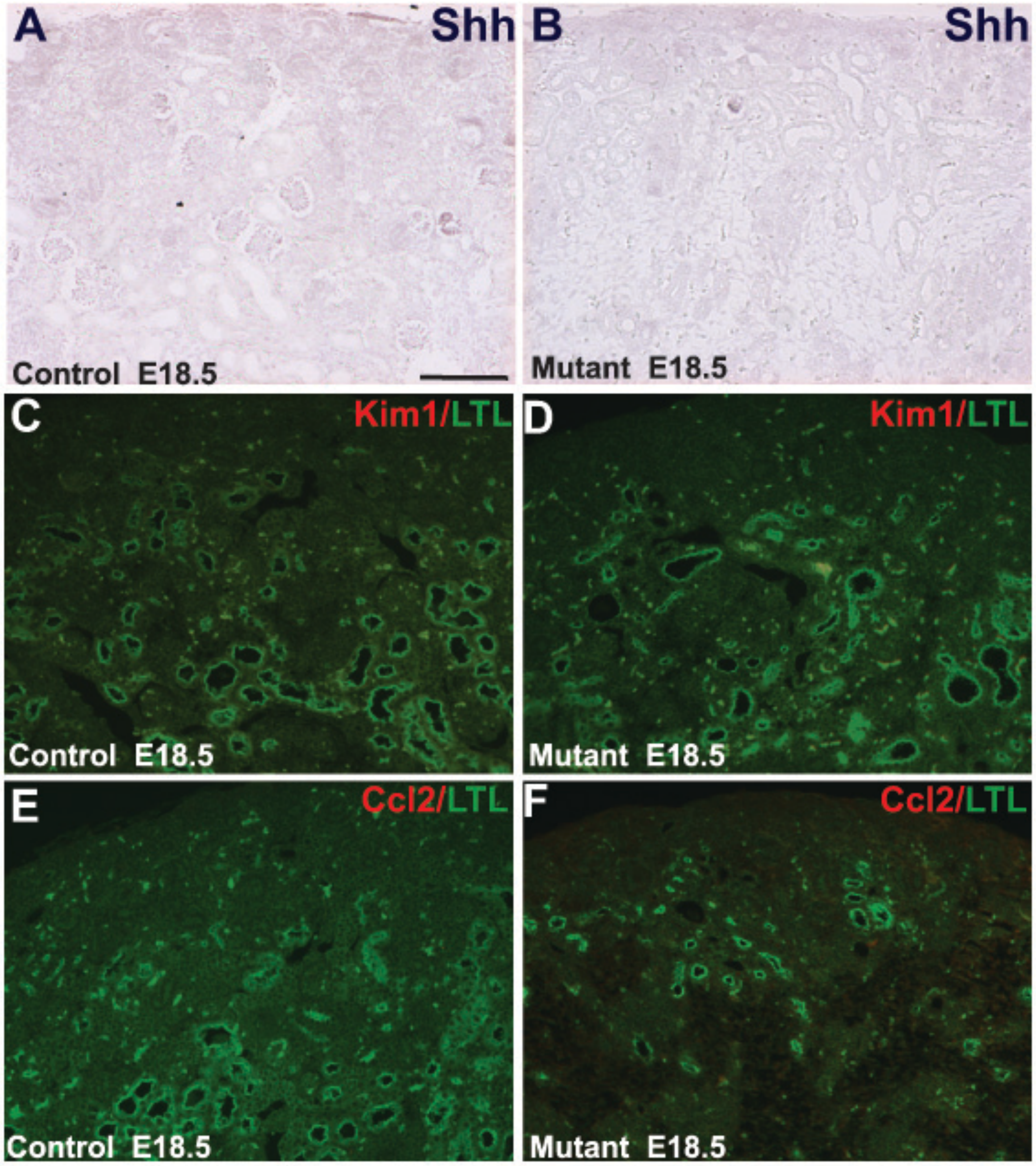

Supplementary Figure 8: E18.5 *Six2creFrs2αKO* kidneys do not display ectopic Shh, Kim1 and Ccl2.

A, B. Immunostaining for Shh in control and mutant E18.5 kidneys (A and B, respectively) reveals no obvious Shh expression. C, D. At E18.5, co-immunofluorescence for LTL (green) and Kim 1 (red) reveals no Kim 1 expression in controls (C) and mutant kidneys (D), including LTL-positive proximal tubules. E, F. At E18.5, co-immunofluorescence for LTL (green) and Ccl2 (red) reveals no Ccl2 expression in controls (E) and mutant kidneys (F), including LTL-positive proximal tubules. A-F scale bar = 100 μm.

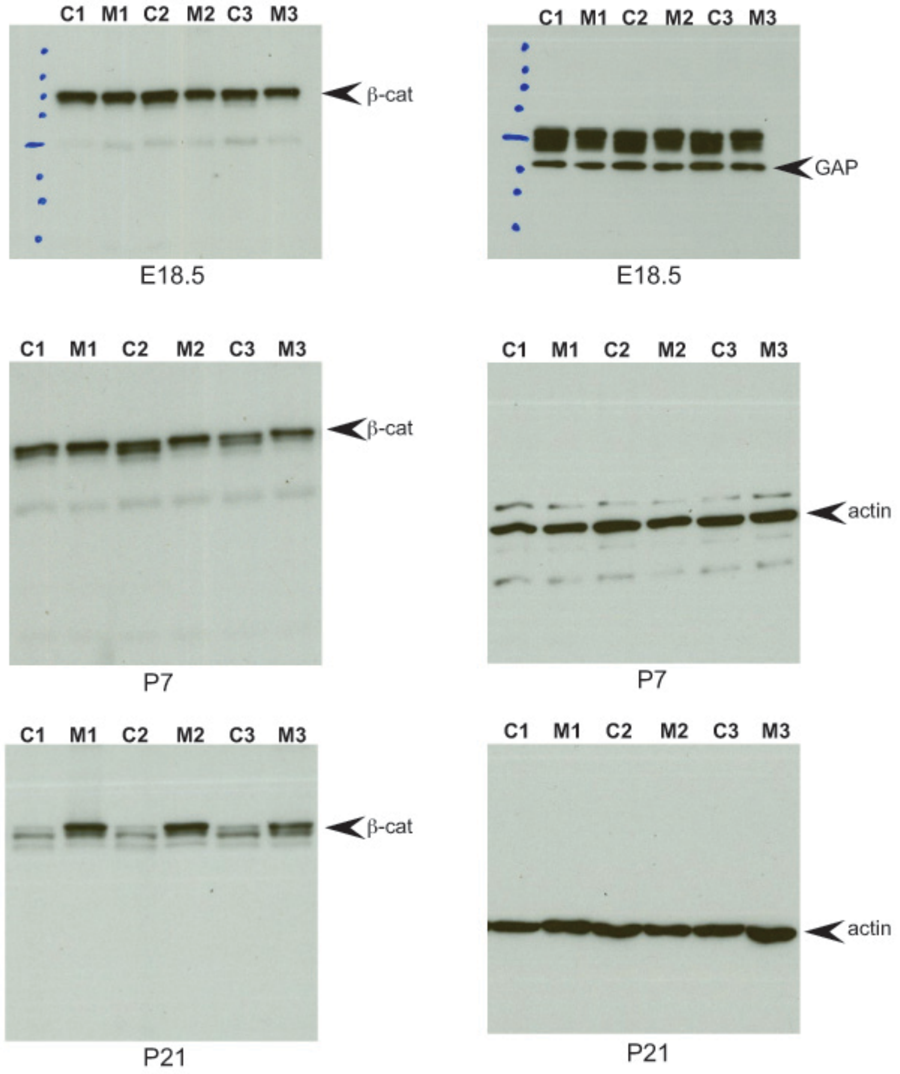

Supplementary Figure 9: Uncropped blots used for the construction of composite of Figure 4G.

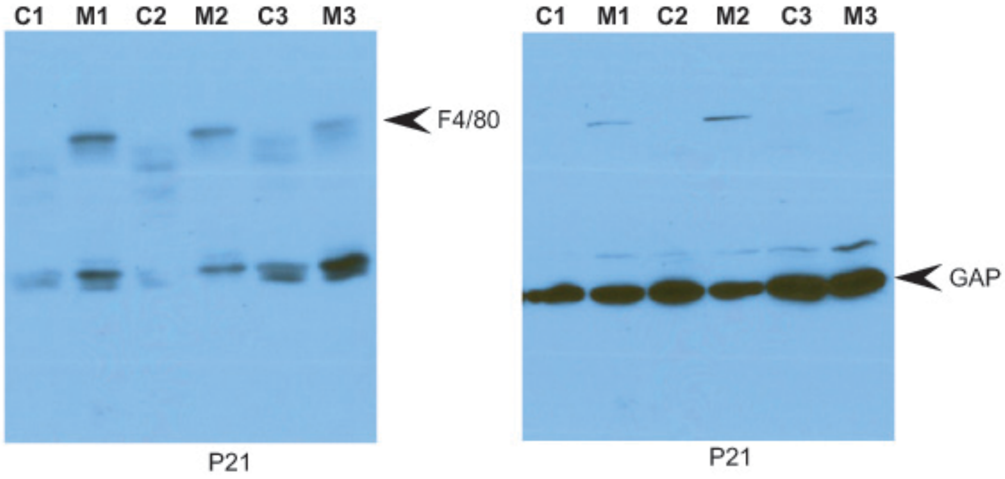

Supplementary Figure 10: Uncropped blots used for the construction of composite of Figure 5B.

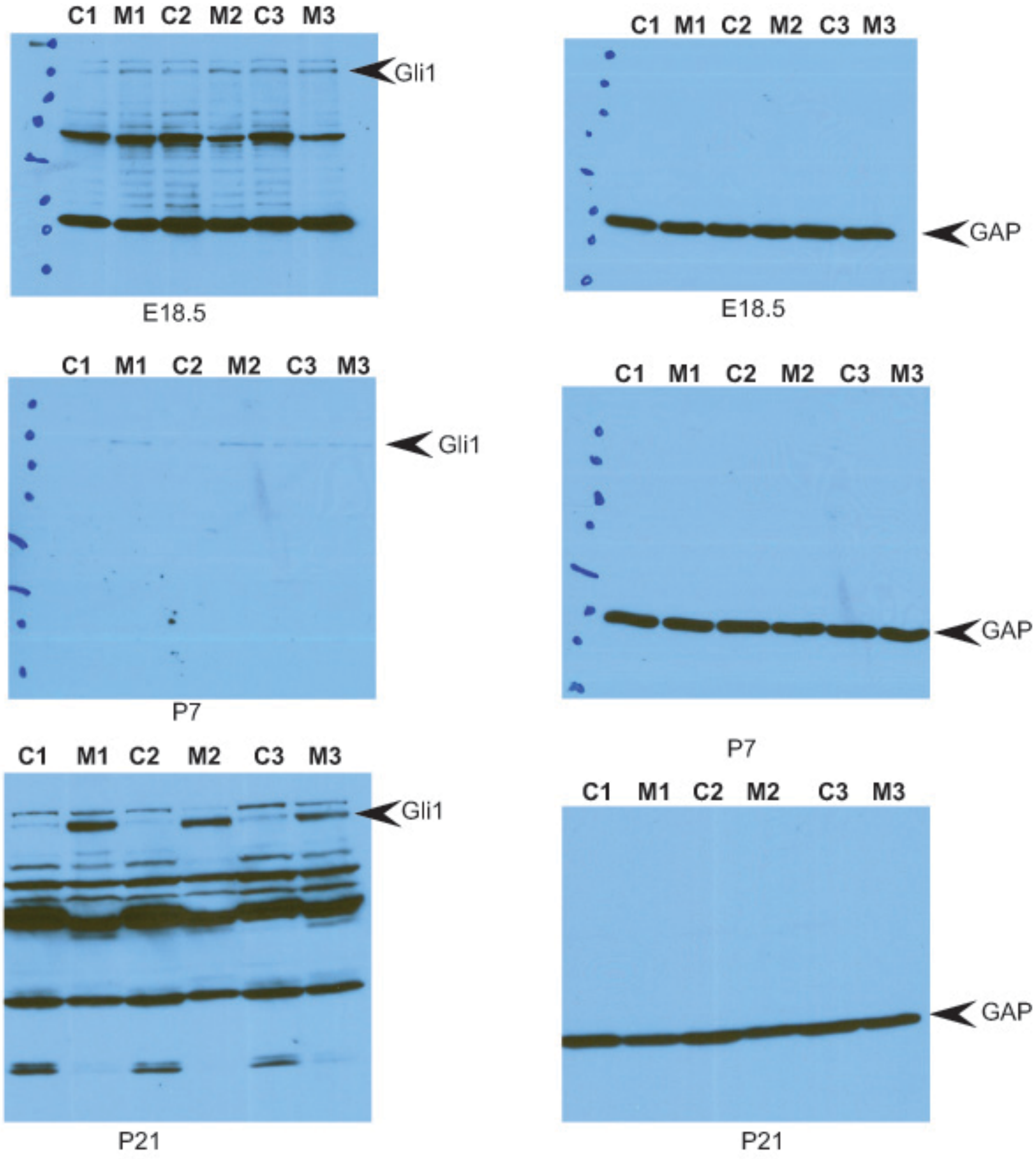

Supplementary Figure 11: Uncropped blots used for the construction of composite of Figure 7B.

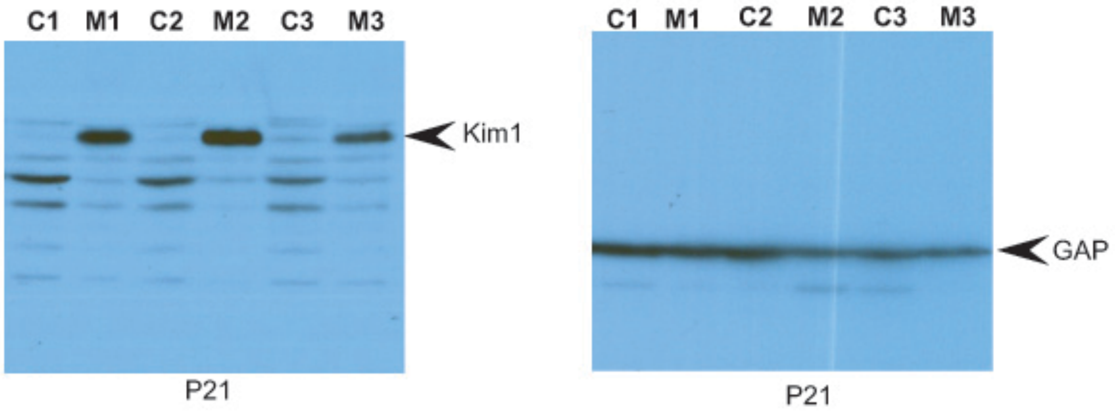

Supplementary Figure 12: Uncropped blots used for the construction of composite of Figure 9B.

Supplementary Tables:

Supplementary Table 1: Antibodies/lectins and dilutions:

Antibody	Company	Catalog #	Application	Dilution
Ki-67	Thermo Scientific	RM-9106-S1	Immunofluorescence	1:1000
αSma	Sigma	A5316	Immunofluorescence	1:1000
β-catenin	BD	610154	Western Blotting	1:5000
F4/80	eBiosciences<br>Thermo Scientific	14-4801-81<br>MA1-91124	Western Blotting<br>Immunofluorescence	1:1000<br>1:1000
Ccl2	Thermo Scientific	MA5-17040	Immunohistochemistry	1:500
Gli1	Novus Biologicals	NB600-600	Western Blotting	1:1000
Shh	Santa Cruz Biotechnology	SC-9024	Immunohistochemistry	1:200
Kim1	Thermo Scientific	PA5-20244	Western Blotting<br>Immunofluorescence	1:1000<br>1: 500
Aquaporin 1	Santa Cruz Biotechnology	SC-9878	Immunofluorescence	1:1000
Acetylated-tubulin	Cell Signaling	5355	Immunofluorescence<br>Immunohistochemistry	1:500<br>1:500
Gapdh	Santa Cruz Biotechnology	SC-32233	Immunofluorescence	1:1000
Fluorescein labeled Lotus Tetragonolobus Lectin (LTL)	Vector Lab	FL-1321	Immunohistochemistry	1:1000
Fluorescein labeled Dolichos Biflorus Agglutinin (DBA)	Vector Labs	FL-1031	Immunohistochemistry	1:1000
Biotinylated labeled LTL	Vector Labs	B-1325	Immunohistochemistry	1:1000
DIG-AP	Roche	11093274910	*in situ* hybridization	1:700

Supplementary Table 2: Qualitative real time PCR primers

Gene Name	NCBI Accession number	sequence	Expected Size (bp)
Acta2	NM_007392	Sense: 5' - GAAGGATCTCTATGCTAACAAC - 3'<br>Anti-sense: 5' - CACATCTGCTGGAAGGTA - 3'	198
Axin2	NM_015732	Sense: 5' - ACAGCATCTTCACCACTT- 3'<br>Anti-sense: 5' - AGAAACCCTCACTTCCTAAA- 3'	154
Ccl2	NM_011333	Sense: 5' – TTTCCACAACCACCTCAAGC – 3'<br>Anti-sense: 5' – GAGGGAAAAATGGATCCACACC – 3'	177
Col1a1	NM_007742	Sense: 5' – TGGCGGTTATGACTTCAG – 3'<br>Anti-sense: 5' – TGCGGATGTTCTCAATCT – 3'	161
Col3a1	NM_009930	Sense: 5' – CTGAAGATGTCGTTGATGTG – 3'<br>Anti-sense: 5' – ACTGTCTTGCTCCATTCC – 3'	247
Cxcl16	NM_023158	Sense: 5' – TTTTCTTGTTGGCGCTGCTG – 3'<br>Anti-sense: 5' – ACACGCTTTTGGACTGCAAC – 3'	195
Cxcl2	NM_009140	Sense: 5' – TGGAAGGAGTGTGCATGTTC – 3'<br>Anti-sense: 5' – TTGCTAAGCAAGGCACTGTG – 3'	188
Dhh	NM_007857	Sense: 5' – ACGAAATCGAAACCGAAGCC– 3'<br>Anti-sense: 5' – AAATTATCCGGTGCGACTGG– 3'	247
Gli1	NM_010296	Sense: 5' – GTCCACCAACCAACTATG – 3'<br>Anti-sense: 5' – TGTCCATAATGCTCAAGTC – 3'	166
Gli2	NM_001081125	Sense: 5' – GCACTATCTGACCACTCT – 3'<br>Anti-sense: 5' – GCCATACTGAACAAGCAA – 3'	247
Gli3	NM_008130	Sense: 5' – CAGTAGGTGTTAGGTAATAGGA – 3'<br>Anti-sense: 5' – CACGGTGAACTTACAGATG – 3'	184
Havcr1	NM_134248	Sense: 5' – AGGAAGTCAGCATCTCTAAGCG – 3'<br>Anti-sense: 5' – ACACAGAAAATCGCCTTGGC – 3'	237
Ift20	NM_018854	Sense: 5' – CCTATGACATACCTCCTGATT – 3'<br>Anti-sense: 5' – CTCAATTAGACCACCAACAAT – 3'	244
Ift52	NM_172150	Sense: 5' – ACTGATGAAGACCTGGAAT – 3'<br>Anti-sense: 5' – TAAGCACTGAAGAACTAATGAAT – 3'	219
Ift88	NM_009376	Sense: 5' – CAACCTCTCGTTCCTGTA – 3'<br>Anti-sense: 5' – GCTGCCTTCTCATAATCAC – 3'	150
Ihh	NM_010544	Sense: 5' – GCGGACAATCATACAGAAC– 3'<br>Anti-sense: 5' – TGTGAGAGGAGCATAGGA – 3'	162
Invs	NM_010569	Sense: 5' – CCACACTCTAACTAAGACTACT – 3'<br>Anti-sense: 5' – TACGATGCCTTCATTCACT – 3'	234
Kif12	NM_010616	Sense: 5' – TTGCAGGCAGTGAGAAAGTG – 3'<br>Anti-sense: 5' – AACTTGGTGAGCTTGCTGTC – 3'	169
Kif3a	NM_008443	Sense: 5' – ACTCGTCGTAATCCTTCTG – 3'<br>Anti-sense: 5' – GGCTTCCTACCTTCTTCAA – 3'	187

Lef1	NM_010703	Sense: 5’ – AGCTTGTTGAAACCCCAGAC– 3’<br>Anti-sense: 5’ – TTTTTGGAAGTCGGCGCTTG– 3’	160
Ocrl	NM_177215	Sense: 5' - TCAAGTGCTGTGTAAGAGA - 3'<br>Anti-sense: 5' - ATCAGATGTTAATAGGCAAGAAT - 3'	249
Ptch1	NM_008957	Sense: 5' - CTGCCTGTCCTCTTATCC - 3'<br>Anti-sense: 5' - CTGCTGTGCTTCGTATTG - 3'	225
Ptch2	NM_008958	Sense: 5' - ACAAGCTGCTCATCCAAACC - 3'<br>Anti-sense: 5' - TTTGTCGTGAAGCCACTCTG - 3'	206
Shh	NM_009170	Sense: 5' - ACCTTCAAGAGCCTTAACT - 3'<br>Anti-sense: 5' - GCATAGCAGGAGAGGAAT - 3'	177
Smo	NM_176996	Sense: 5' - CCGCACTAACCTAATGGA - 3'<br>Anti-sense: 5' - AATCGCTGTATTCAACTTGTT - 3'	230
Tnf	NM_013693	Sense: 5' - CCAGTCTGTATCCTTCTAACT - 3'<br>Anti-sense: 5' - ATCTTATCCAGCCTCATTCT - 3'	250
Tnfaip6	NM_009398	Sense: 5' - ACATGCAAAGGAGTGTGGTG - 3'<br>Anti-sense: 5' - TCTTCCTACAAAGCCGTGGAC - 3'	235
Wnt4	NM_009523	Sense: 5' - TCTCTGCTCATTGTCCAT - 3'<br>Anti-sense: 5' - TGCTGAACTAAGTCTACCA - 3'	179
Wnt7b	NM_009528	Sense: 5' - GCCAATCTTCCATTCCATT - 3'<br>Anti-sense: 5' - CCTCTGTCCATCTGTCAT - 3'	230
Wnt9b	NM_011719	Sense: 5' - CTTGAAGTTGAGGCTGAG - 3'<br>Anti-sense: 5' - ATGTATGAGGTAGGCAGAA - 3'	204
